# Empowering pregnant women to improve diet quality: a RCT among pregnant women in the Netherlands

**DOI:** 10.1093/eurpub/ckac131.442

**Published:** 2022-10-25

**Authors:** RM van Lonkhuijzen, A Wagemakers, JHM de Vries

**Affiliations:** 1 Health and Society, Wageningen University and Research, Wageningen, Netherlands; 2 Human Nutrition and Health, Wageningen University and Research, Wageningen, Netherlands

## Abstract

**Objective:**

A healthy diet during pregnancy is crucial for the health of both mother and child, but pregnant women often do not meet the nutritional requirements. Empowering pregnant women to improve their diet quality could play a significant role in improving maternal nutrition and health. However, empowerment has been rarely used in nutritional interventions. Based on research and input from stakeholders, we developed Power 4 a Healthy Pregnancy (P4HP). P4HP consists of four additional consults by the midwife or dietician to discuss nutrition with the pregnant women from an empowerment perspective. This study aims to evaluate the effectiveness of P4HP on diet quality, empowerment, and health of pregnant women.

**Methods:**

A cluster randomized controlled trial started in January 2022 in 14 Dutch midwifery practices, with a total of 175 pregnant women in both the control and intervention groups (N = 350). Women in the intervention group follow P4HP in addition to their usual birth care. Measurements are carried out at the beginning and end of pregnancy. Diet quality is measured using the Dutch Healthy Diet index 2015, specifically adapted for pregnancy. Empowerment is assessed using the Pregnancy-Related Empowerment Scale, Sense of Coherence (SOC) using the SOC-3 scale, Self-Rated Health using a General Self-Rated Health question, and Quality of Life using a Visual Analogue Scale. Results will be analyzed using Linear Mixed Models to analyze the treatment effect of clustered data.

**Results:**

Baseline results of 100 pregnant women indicate that they do not meet the Dutch dietary guidelines. We hypothesize that P4HP will lead to improvements in diet quality, empowerment, and health among the intervention group compared to the control group.

**Conclusions:**

Our findings will show the effect of P4HP on pregnant women’s diet quality, empowerment, and health. We expect to present our preliminary results during the congress.

**Key messages:**

• Our findings will show the effect of four additional nutritional consultations from an empowerment perspective on pregnant women’s diet quality, empowerment and health.

• Empowering pregnant women to improve their diet quality could play a significant role in improving maternal nutrition and health.

